# Genetic and pharmacological inhibition of TTK impairs pancreatic cancer cell line growth by inducing lethal chromosomal instability

**DOI:** 10.1371/journal.pone.0174863

**Published:** 2017-04-05

**Authors:** Jeran K. Stratford, Feng Yan, Rebecca A. Hill, Michael B. Major, Lee M. Graves, Channing J. Der, Jen Jen Yeh

**Affiliations:** 1 Department of Pharmacology, University of North Carolina at Chapel Hill, Chapel Hill, North Carolina, United States of America; 2 Lineberger Comprehensive Cancer Center, University of North Carolina at Chapel Hill, Chapel Hill, North Carolina, United States of America; 3 Department of Cell Biology and Physiology University of North Carolina at Chapel Hill, Chapel Hill, North Carolina, United States of America; 4 Department of Computer Science, University of North Carolina at Chapel Hill, Chapel Hill, North Carolina, United States of America; 5 Department of Surgery, University of North Carolina at Chapel Hill, Chapel Hill, North Carolina, United States of America; University of Texas Health Science Center at San Antonio, UNITED STATES

## Abstract

Pancreatic ductal adenocarcinoma, which accounts for the majority of pancreatic cancers, is a lethal disease with few therapeutic options. Genomic profiling of pancreatic ductal adenocarcinoma has identified a complex and heterogeneous landscape. Understanding the molecular characteristics of pancreatic ductal adenocarcinoma will facilitate the identification of potential therapeutic strategies. We analyzed the gene expression profiles of primary tumors from patients compared to normal pancreas and identified high co-overexpression of core components of the spindle assembly checkpoint, including the protein kinase TTK (also known as MPS-1). We found overexpression of TTK protein in a subset of pancreatic ductal adenocarcinoma primary tumors and cell lines. siRNA-mediated depletion or catalytic inhibition of TTK resulted in an aberrant cell cycle profile, multi- and micro-nucleation, induction of apoptosis, and decreased cell proliferation and transformed growth. Selective catalytic inhibition of TTK caused override of the spindle assembly checkpoint-induced cell cycle arrest. Interestingly, we identified ubiquitin specific peptidase 16 (Usp16), an ubiquitin hydrolase, as a phosphorylation substrate of TTK. Usp16 regulates chromosomal condensation and G2/M progression by deubiquitinating histone H2A and polo-like kinase 1. Phosphomimetic mutants of Usp16 show enhanced proteosomal degradation and may prolong the G2/M transition allowing for correction of replication errors. Taken together, our results suggest a critical role for TTK in preventing aneuploidy-induced cell death in pancreatic cancer.

## Introduction

Pancreatic ductal adenocarcinoma (PDAC) represents 85% of all pancreatic cancers [1] and is projected to be the third leading cause of cancer related deaths in the United States in 2016 [2]. Median survival of pancreatic cancer patients is five to eight months with fewer than 5% of patients surviving longer than five years after diagnosis. The poor prognosis stems from the frequent presence of metastatic disease at the time of or shortly after diagnosis. The current standard of care for metastatic pancreatic cancer is chemotherapy. Although chemotherapeutic approaches including gemcitabine, nab-paclitaxel, and FOLFIRINOX have improved patient survival [3–5], the discovery of new and better drug targets remains essential for the continued improvement of therapies for PDAC.

Genomic and mouse model studies have advanced our understanding of PDAC tumor biology and have identified a high degree of chromosomal instability in PDAC [6–8]. One aspect of chromosomal instability is the unequal segregation of chromosomes during mitosis, resulting in aberrant chromosomal numbers and cellular aneuploidy of both daughter cells [9]. It has long been postulated that chromosomal instability is an important mechanism for tumor adaptation [10,11]. However, recent studies have hypothesized that the adaptive capacity of cancer cells to aneuploidy is limited [12,13]. Aneuploid cancer cells must maintain a delicate balance between sustaining an altered genome that enhances proliferation yet confines continued chromosomal instability within survivable limits [14,15]. As such, there is a pressing need to characterize the adaptive mechanisms that control this balance to potentially identify therapeutic targets that can shift this balance from sustainable to non-viable chromosomal instability.

Chromosomal segregation during mitosis is a multi-step process and errors during segregation often result in aneuploidy [16]. Therefore, the timing of each step is tightly regulated to maintain high levels mitotic fidelity. In normal cells, mitotic checkpoints ensure that each step is completed prior to continuing through the next phase of the cell cycle. The importance of these checkpoints in cancer is an area of current interest as inactivation of mitotic checkpoints has been shown to enhance chromosomal instability [17], which may result in aneuploidy and decreased cellular fitness.

The spindle assembly checkpoint (SAC) is a conserved mitotic checkpoint found from yeast to mammals that ensures accurate segregation of chromosomes during mitosis. During metaphase, sister chromatids congregate at the metaphase plate prior to separation during anaphase. The SAC is activated to prevent the premature onset of anaphase until bi-oriented microtubule attachment occurs at each kinetochore. Failure to activate the SAC has previously have been shown to promote chromosomal instability [9,14,15,18].

SAC activation requires the expression and activity of the protein kinase TTK, also known as Mps1/Pyt/CT96 [19]. Overexpression of TTK has been proposed to be an adaptive mechanism whereby cells cope with aneuploidy. In agreement with this hypothesis, high levels of *TTK* mRNA have been observed in multiple cancer types and been shown to be protective against aneuploidy [20–26]. Previous studies have investigated the role of TTK in cancer using pharmacologic inhibitors in cancer cell lines. Pharmacologic inhibition of TTK in colorectal and glioblastoma cancer cell lines reduced cell viability, caused aberrant cell cycle progression, increased aneuploidy, and increased apoptosis [27–31]. Similarly, two recent studies provide a tantalizing rationale to elucidate the role of TTK overexpression in PDAC. Slee et al. found that pharmacologic inhibition of TTK in PDAC caused aberrant override of SAC-dependent cell cycle arrest, increased chromosomal instability, increased apoptosis and decreased clonogenic survival [32]. Kaistha et al. found that genetic depletion of TTK increased apoptosis and decreased PDAC cell line proliferation and was associated with high levels of chromosomal instability [33]. These studies clearly demonstrate the importance for TTK in continued PDAC growth, yet several questions remain unanswered about the biological role of TTK in PDAC. First, the efficacy of alternative TTK selective inhibitors with differing specificity profiles has not been characterized. Second, the consequences of acute suppression of TTK function on PDAC growth remain undetermined. Third, the phosphorylation substrates required for TTK-dependent growth remain poorly characterized. Finally, the requirement of TTK for cellular transformation and *in vivo* tumorigenesis in PDAC has not been evaluated. In addition, these and other studies clearly implicate the importance of TTK catalytic activity for continued PDAC cell line growth, yet how TTK promotes growth remains unclear. The list of identified TTK phosphorylation substrates remain sparse and the key substrates for TTK-dependent cancer growth remain unestablished [34–37].

The overall goal of this study was to investigate the role of TTK in mitotic progression, proliferation and transformation of PDAC and identify the molecular mechanism whereby TTK limits chromosomal instability. We found increased mRNA expression of core SAC components and elevated mRNA and protein expression of TTK in a subset of PDAC cell lines and human tumors, suggesting that TTK might protect cells from excessive chromosomal instability through SAC activation. We also found that TTK phosphorylates and regulates the protein stability of Usp16, an enzyme required to promote chromosomal condensation, suggesting that TTK functions at multiple stages of the cell cycle to maintain genome stability. Together these findings help explain the role of TTK in adapting to and maintaining viable levels of aneuploidy in PDAC.

## Materials and methods

### Tissue collection

After approval from the University of North Carolina at Chapel Hill Institutional Review Board, de-identified tissue samples were obtained from the UNC Tissue Procurement Core Facility. Consent was not obtained because no identifiers were available to the investigators and the data was analyzed anonymously. Flash-frozen human tumor tissue samples were homogenized in NP-40 lysis buffer, resolved by SDS—PAGE, and evaluated by western blot analysis.

### RNA microarray analysis

Gene expression microarray data of 132 normal and primary pancreatic tumor samples from patients were obtained from the Gene Expression Omnibus (GEO) database (accession number GSE21501). Normalization, quality control, and imputation of array data were performed as previously described [38]. Expression data from multiple probes were collapsed by the mean for each sample. Statistical significance was assessed using a two-tailed unpaired t-test comparing SAC component expression in normal versus tumor tissue. Differentially expressed genes were identified using statistical analysis of microarrays (SAM) and a 5% false discovery rate (FDR) [39]. Gene networks were investigated for enrichment of differentially expressed kinases using Ingenuity Pathway Analysis (IPA, Ingenuity Systems).

### Cell culture and reagents

All cell lines except HPDE, HPNE, 293FT, HuPT3 were obtained from American Type Culture Collection (ATCC, LGC Promochem). The HPV16-E6-E7 immortalized human HPDE cell line was obtained from Ming-Sound Tsao (University of Toronto, Toronto) [40]. The HuPT3 cell line was obtained from Dan Billadeau (Mayo Clinic, Rochester, MN). 293FT cells were purchased from Invitrogen. The HPV16-E6E7-immortalized human HPNE cell line was obtained from Michel Ouellete (UNMC Eppley Cancer Center) and has been described previously [41,42]. All cell lines were maintained at 37°C and 5% CO_2_. HPAC, PANC-1, MIA PaCa-2, T3M4, HPAF-II, HPNE, HPDE, 293T, 293FT, and HeLa cell lines were cultured in Dulbecco’s Modified Eagle Medium (DMEM, Corning). BxPC-3, Panc 02.03, Panc 10.05, AsPC-1, SW-1990, HuPT3, CFPAC-1, Capan-1 and Capan-2 cell lines were maintained in RPMI-1640 with 4.5 g/L glucose. All growth media were supplemented with 10% (vol/vol) fetal bovine serum (FBS, Hyclone), and 100 U/ml penicillin and 100 U/ml streptomycin (P/S, Gibco). Capan-1 cells which were supplemented with 15% (vol/vol) FBS.

### Antibodies

Primary antibodies used for immunoblot or immunoprecipitation include: Mouse anti-β-actin (IB, 1:5000, AC-15, Sigma Aldrich), mouse anti-TTK (IB, 1:2000, 4-112-3 Millipore), rabbit anti-Cyclin B1 (IB, 1:1000, 4138 Cell Signaling), rabbit anti-Histone H3 phospho Ser 10 (IB, 1:1000, 9701 Cell Signaling), mouse anti-FLAG (IB, 1:1000, M2 Sigma Aldrich), mouse anti-phospho tyrosine (IB, 1:1000, 9411 Cell Signaling), mouse anti-phospho serine (IB, 1:1000, 4A4 Millipore), mouse anti-phospho threonine (IB, 1:1000, Cell Signaling 3986) rabbit anti-Usp16 (IB, 1:2000, Bethyl A301-615A), mouse anti-HA (1:1000, Covance 16B12), and mouse anti-green fluorescent protein (GFP) (1:5000 Clontech JL8). HRP conjugated secondary antibodies included goat anti-mouse (1:10,000, Thermo) or goat anti-rabbit (1:10,000, Thermo).

### Immunoprecipitation and immunoblotting

Two to five μg of specified antibodies were bound to 20–50 μl of Protein G Dynabeads (Invitrogen) overnight at 4°C. Cells were collected following treatments as specified in the figure legends. Cells were harvested and then lysed in NP-40 lysis buffer (50 mM Tris pH 7.5, 10 mM MgCl_2_, 150 mM NaCl, 1% NP-40, 0.25% sodium deoxycholate, and 10% glycerol) supplemented with phosphatase (Roche) and protease (Sigma) inhibitor cocktails. Cell lysates were incubated with antibody bound Dynabeads overnight at 4°C. Beads were washed three times with lysis buffer and resuspended in protein sample buffer containing β-mercaptoethanol and examined by immunoblotting.

Immunoblots were performed by running protein samples on either 15% (for proteins under 40 kDa) or 8% SDS-PAGE followed by transfer of resolved proteins to PVDF membranes for 90 min at 4°C. All immunoblots were blocked in 5% milk/TBST solution for 1 h at room temp with gentle agitation. Primary antibodies were diluted in 5% (weight/vol) milk or 5% BSA. Primary antibodies were incubated overnight at 4°C. Following incubation with primary antibody, blots were washed with TBST and then incubated with secondary HRP-conjugated antibodies for 1 h at room temp with gentle agitation. Blots were again washed with TBST and developed by ECL/chemiluminescence and autoradiograph film (Kodak) or the Biorad XRS+ imaging system.

FLAG pull-downs were performed on cell lysates using the pull-down buffer (50 mM Tris pH 7.6, 150 mM NaCl, 1% NP-40, 0.25% sodium deoxycholate, 1 mM EDTA, 1 mM EGTA) and supplemented with protease inhibitors (Roche). Lysates were clarified by centrifugation. Ten mg of protein were used in each pull down. Anti-FLAG M2 magnetic beads (Sigma) were used to immunoprecipitate FLAG-tagged Usp16. Following extensive washing with TBS, FLAG-Usp16 was eluted with FLAG peptide (Sigma) in TBS.

### cDNA expression

The pLenti CMV Puro LUC (w168-1) expression vectors for firefly luciferase was a gift from Eric Campeau (Addgene plasmid #17477) [43], Flag-HA-USP16 was a gift from Wade Harper (Addgene plasmid # 22595) [44]. GFP-Usp16 was prepared by amplifying Usp16 cDNA from FLAG-HA-Usp16 followed by subcloning into pEGFP-C3 using the XhoI and BamHI sites. GFP-Usp16 3xA and 3xE were prepared by site-directed mutagenesis and confirmed by sequencing.

Transfections for all cDNA were accomplished using an HBS/CaCl_2_ or TransIT-LT1 (Mirius Bio) protocol and cells were examined for expression 48–72 h post transfection

### shRNA constructs and infection

pLKO.1 shRNA lentiviral plasmids were obtained from the UNC lentiviral RNAi core facility including: shTTK_3 (TRCN0000006358) and shTTK_4 (TRCN0000011011).

To establish stably infected pancreatic cells expressing shRNA, pLKO.1 lentiviral vector was transiently transfected into 293T cells with the pCMV-VSV-G (Addgene plasmid #8454), pMDLg/pRRE (Addgene plasmid #12251) and pRSV-Rev (Addgene plasmid #12253) packaging plasmids using HBS/CaCl_2_ transfection reagent. Viral supernatants were collected and infection of PDAC cell lines was performed in growth media supplemented with 8g/ml polybrene. Cells stably expressing the shRNA were selected by culturing the cells in media containing 1.0–2.0 μg/ml puromycin. Following selection, cells were maintained in puromycin at one-half the concentration used for selection.

### siRNA transfection

All siRNA described were obtained from Thermo and are part of the ON-TARGETplus SMARTpools of siRNA. siRNAs were transfected using RNAiMax (Invitrogen).

### Cell proliferation assays

For the MTT viability assay, 3 x 10^5^ cells were transfected with siRNA using RNAiMax transfection reagent. Forty-eight h post transfection 1 x 10^3^ cells were plated into 6 identical wells of a 96-well plate. Proliferation was assessed by addition of the MTT reagent for 4 h. Measurements were taken at A_560_ on the Synergy 2 (Biotek Instruments).

For the soft agar colony formation assay, 10^4^ siRNA-transfected cells were suspended between layers of 0.6% (bottom) and 0.3% (top) bacto-agar (BD Biosciences) in three wells of a 6-well dish. Cells were supplied with growth medium twice a week and allowed to form colonies for 7–28 days. Colonies were visualized by staining with MTT (2 mg/ml). Colonies were imaged and quantitated using ImageJ (National Institute of Health).

### Cell cycle analysis by flow cytometry

DNA content was assayed using propidium iodide. Cells were harvested, washed and fixed in 70% ethanol. Cells were permeabolized with 0.5% Triton X-100 and stained with propidium iodide. Stained cells were quantitated on the CyAN flow cytometer (Beckman Coulter). Distribution of cells in each stage of the cell cycle was performed using ModFit (Verity Software House).

### Immunofluorescence

PANC-1 and HPAC cells were plated on poly-L-lysine (Sigma) coated coverslips. Cells were fixed in 4% paraformaldehyde, permeabolized with 0.2% Triton X-100. Cells were then blocked in 5% BSA in PBS. Cells were allowed to stain for 2 h at room temperature, washed three times with PBS. Alexa Fluor-conjugated secondary antibodies (Invitrogen) and DAPI were allowed to bind for 1 h. Stained cells were washed three times with PBS, followed by one wash with distilled water. Coverslips were then mounted with Fluorsave (Calbiochem).

All confocal images were obtained on a Zeiss 710 spectral confocal laser scanning confocal microscope equipped with a 405, 458, 488, 514, 543, 594, 633 nm excitation lines. Images were obtained using a 40X or 63X oil plan/Apo objective. Multicolor images were acquired using sequential scanning. All images were visualized using ImageJ and cropped using Adobe Photoshop version CS3.

### Apoptosis evaluation

Apoptosis was evaluated using an Annexin V assay (Roche) following the manufacturer’s protocol. Live cells were harvested in Tryp-LE (Gibco), washed in PBS, incubated in binding buffer with FITC conjugated Annexin V. Necrotic cells were discriminated by using a propidium iodide counterstain. Stained cells were quantitated on the CyAN flow cytometer (Beckman Coulter). At least 10,000 events were analyzed for each sample.

The Caspase-Glo 3/7 Assay kit (Promega, Madison, WI) was used to measure apoptotic behavior in cells following the manufacturer’s protocol. Briefly, cells were transfected with siRNA and 100 μL was transferred to a 96-well tissue culture plate with black walls and clear bottom. Cells were incubated for 72 h. For drug treated cells, cells were given 24 h to adhere to the plate prior to treatment. Cells were then treated with 2 μM AZ3146 (72 h), 0.1% DMSO (24 h), or 2 μM staurosporine (24 h) in 100 μL of media. Luminescence substrate for caspase 3/7 (100 μL) was added to each well and gently mixed. The samples were incubated for 90 min in the dark at room temperature and luminescence was measured in a Synergy 2 plate reader (BioTek Instruments) as directed by the manufacturer. Each treatment group is represented in triplicate.

### P^32^ incorporation assay

Kinase assays were performed using recombinant active GST-tagged TTK (SignalChem). FLAG-Usp16 was purified from 293FT cells stably expressing FLAG-Usp16. Kinase assay was performed at 30°C for 15 and 30 min in 50 μl assay buffer (5 mM MOPS pH 7.2, 2.5 mM beta glycerol phosphate, 5 mM MgCl_2_, 1 mM EGTA, 0.4 mM EDTA, 200 μM ATP, 0.4 mCi/ml [ϒ-^32^P]ATP, 50 μM DTT, 50 ng/μl BSA). Five microliters of the reaction was spotted onto Whatman P81 cellulose phosphate filter paper. The filter paper was washed with 10% phosphoric acid three times for 5–10 min each to quench the reactions. Filter paper was washed once with ethanol and dried. Filter papers were placed into scintillation vials, scintillation fluid was added to each vial, and ^32^P incorporation was assessed on the Beckman LS6500 (Beckman Coulter).

### Phosphopeptides enrichment and mass spectrometry

Following trypsin digestion with FASP protocol using spin filter column (Sartorius Vivacon 500 30kD cutoff), the peptides mixture was reduced to about 100 μl by vacuum centrifugation. Titanium dioxide tips (GL Sciences Phos-TiO Kit, 1404024) was used to enrich phosphopeptides afterwards. In brief, the peptides mixture was loaded to the TiO_2_ tip first. After equilibration and washing, phosphopeptides were eluted off the tips with 50 μl 5% ammonium hydroxide solution (Fisher Chemical Optima grade, A470-250) and 50 μl 5% pyrrolidine solution (Acros Organics, AC44614-1000). The eluate was concentrated to a volume of 5 μl or less in SpeedVac concentrator, and reconstituted with 0.1% FA water for mass spectrometric analysis (Thermo Orbitrap Elite mass spectrometer).

## Results

### High expression of spindle assembly checkpoint kinases identified in PDAC

Mutations in protein kinases underlie many human diseases, including cancer, and previous success of FDA approved kinase inhibitors make protein kinases an attractive class of highly druggable molecular targets [45]. However, the percent of the kinome with at least one variations that affects protein coding (missense/deletion/frame shift) is lower in PDAC (75%) compared to breast (94%), lung (86%), and colon (93%) cancers [46–49] (https://tcga-data.nci.nih.gov/). Gene amplifications and overexpression of protein kinases has also been shown to promote oncogenesis [50]. Thus we examined which protein kinases are overexpressed in PDAC by analyzing gene expression profiles of previously published cDNA microarrays of primary PDAC tumors (n = 30) and normal pancreas (n = 20) [38]. We identified differentially expressed kinases using the Statistical Analysis of Microarrays (SAM) software [39,51]. Of 3899 differentially expressed genes between primary patient tumors and normal pancreas, 106 probes representing 91 kinases were identified (S1 Table).

**Fig 1:**
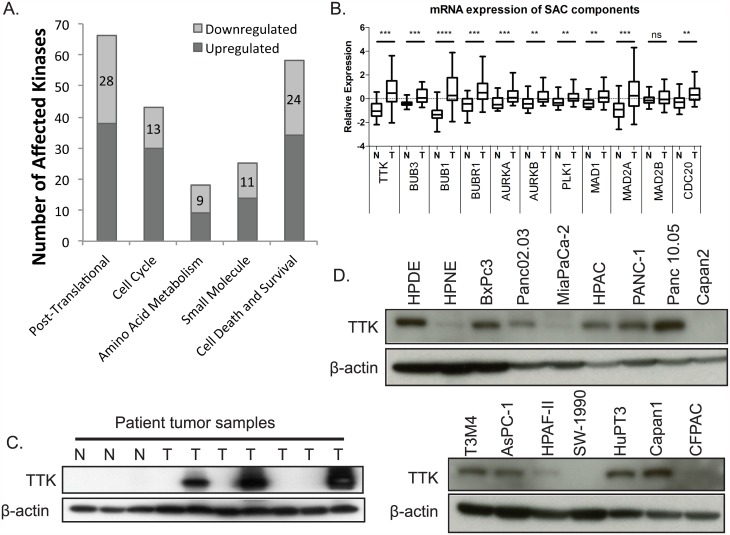
Increased expression spindle assembly checkpoint components and its regulator TTK in PDAC. (A) IPA software identified prominent cellular functions that were significantly affected by differentially expressed kinases between normal and primary PDAC. (B) Box and whisker plot of median, upper, and lower quartiles of mRNA expression of core components and regulators of the spindle assembly checkpoint. Asterisk represent the p-value of the Mann-Whitney test (ns: p≥0.05, **: p≤0.01, ***: p≤0.001, ****: p≤0.0001). (C) Expression of TTK in a panel of patient samples. N = normal pancreas and T = primary tumor. (D) Expression of TTK in immortalized pancreas epithelium (HPNE and HPDE) and PDAC cell lines.

**Fig 2:**
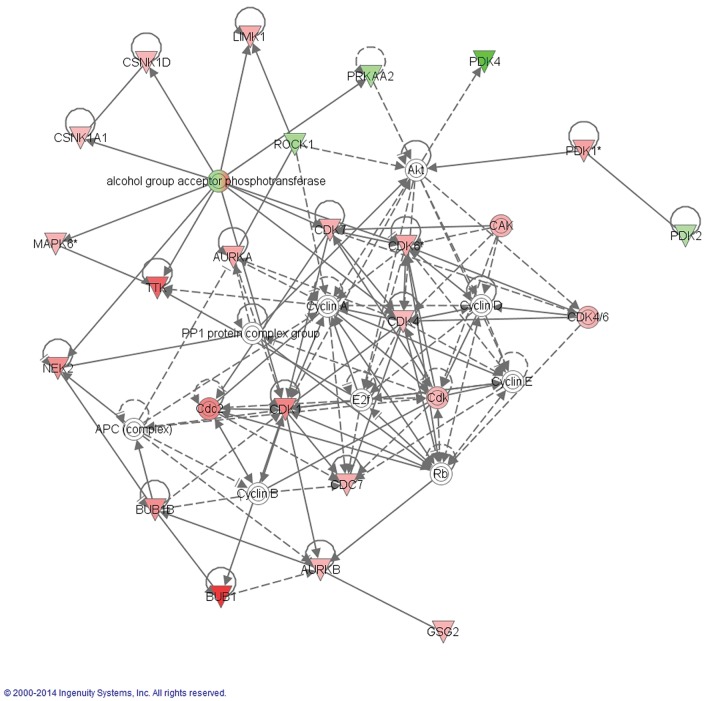
Ingenuity pathway analysis of differentially expressed kinases in primary PDAC compared to normal pancreas identified by unbiased methods. All 106 differentially regulated kinases were analyzed by integrated pathway analysis using IPA. The posttranslational modification cell cycle, cellular assembly and organization, one of the most dominant networks, is depicted here. Signaling pathways are colored according to expression, green representing down-regulation and red representing up-regulation with the expression fold change represented by more intense colors.

We identified biological networks regulated by the differentially expressed kinases using IPA. We found that the cell cycle was one of the most enriched biological pathways regulated by our list of kinases in PDAC (Table 1, Figs 1A and 2), with 7 of the 10 most up-regulated kinases (Table 2). Interestingly, three of these seven kinases are involved with the spindle assembly checkpoint (SAC): BUB1, BUB1B, and TTK. Further interrogation of the gene expression profiles found statistically significant co-overexpression of both kinase and non-kinase components of the SAC in PDAC tumors compared to normal pancreas (Fig 1B). Together these data suggest that the SAC may be important for mitotic fidelity in PDAC.

**Table 1:** Biological pathways with enrichment of differentially expressed kinases.

Network name	*p-*value	# of nodes
Post-translational modification	1.14E-67–3.85E-04	66
Cell cycle	6.76E-24–4.16E-04	43
Amino acid metabolism	1.46E-19–1.45E-08	18
Small molecule biochemistry	1.46E-19–2.42E-06	25
Cell death and survival	5.53E-19–4.16E-01	58

**Table 2:** Function of the 10 most overexpressed kinases.

Kinase	Fold Change	FDR (q-value)	Function
BUB1	3.887	0	Cell cycle
NEK2	3.424	0	Cell cycle
CHEK1	3.362	0	Cell cycle
PTK6	3.213	0	Differentiation and apoptosis
TTK	2.972	0	Cell cycle
PBK	2.756	0	Cell cycle
STYK1	2.65	0.125	Proliferation and survival
MET	2.381	0	Growth factor sensing
CDK1	2.343	0	Cell cycle
BUB1B	2.114	0	Cell cycle

TTK kinase activity is essential to activate the SAC and prevent early anaphase onset [52–55]. In order to evaluate the role of TTK we evaluated the expression of TTK at the protein level and confirmed that a subset (43%) of primary PDAC patient samples had elevated TTK protein expression compared to normal pancreas tissue (Fig 1C). In addition, we evaluated TTK protein expression in a panel of 14 PDAC cell lines by immunoblot analysis (Fig 1D). Five cell lines were selected with variable TTK expression for further investigation: HPAC, SW-1990, AsPC-1, Panc 10.05 and PANC-1.

### TTK is necessary for PDAC growth

**Fig 3:**
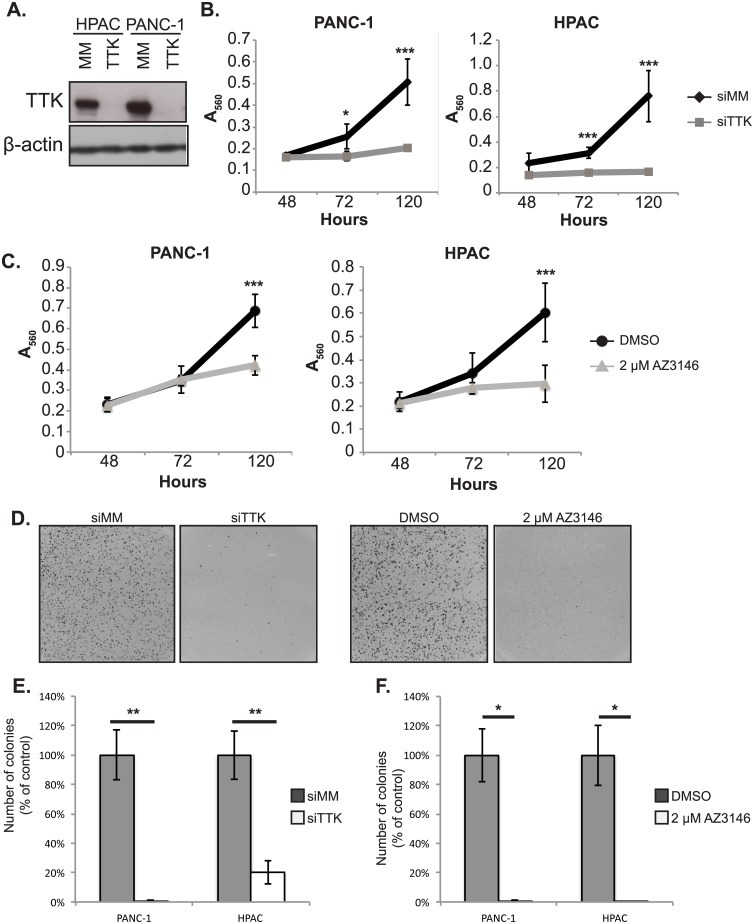
Genetic and pharmacologic inhibition of TTK decrease growth of PDAC cell lines. (A) Immunoblot analysis of HPAC and PANC-1 cell extracts showing protein level of TTK in control mismatch siRNA (siMM) and a TTK siRNA (siTTK) pool 48 h after transfection. (B) Growth of HPAC and PANC-1 PDAC cell lines transfected with control siMM and siTTK show reduced viability with TTK depletion. Cells were measured for proliferation at 48, 72, and 120 h as indicated. (C) Growth of HPAC and PANC-1 PDAC cell lines treated with DMSO control or 2 μM AZ3146. Cells were measured for proliferation at 48, 72, and 120 h as indicated. (D) Representative images of colony formation of the PANC-1 cell line in soft agar. (E) Quantitation of colony formation in soft agar of the HPAC and PANC-1 cell lines after transfection of either control or *TTK* targeted siRNA. Samples normalized to control. (F) Quantitation of colony formation in soft agar of the HPAC and PANC-1 cell lines after with continuous treatment with vehicle (DMSO) or AZ3146. Normalized to DMSO control. Asterisk represent the P-value of the two-sided T-test (*:≤0.05, P **: P≤0.01). Results representative of at least 2 independent experiments.

To determine the functional consequence of TTK expression on PDAC growth we depleted TTK in PDAC cell lines using a pool of 5 individually targeted siRNAs (S1 Fig). TTK protein knockdown was confirmed by immunoblot analysis in four PDAC cell lines: AsPC-1, HPAC, PANC-1, and Panc 10.05 (Fig 3A, S2 Fig panel A). Depletion of TTK in all four cell lines decreased growth on plastic as determined by the thiazolyl blue tetrazolium bromide (MTT) assay (Fig 3B, S2 Fig panel B). Catalytic inhibition of TTK with the AZ3146 TTK-selective protein kinase inhibitor (AstraZeneca) phenocopied the decrease in proliferation seen with siRNA silencing of TTK expression in HPAC and PANC-1 cells with a less dramatic effect in Panc 10.05 cells. However, catalytic inhibition of TTK had no effect on AsPC-1 proliferation despite equivalent drug administration (Fig 3C, S2 Fig Panel C).

We next examined the requirement for TTK to support PDAC transformed growth by measuring anchorage-independent colony formation in soft agar, a standard assay for cellular transformation. PANC-1 and HPAC cells with either genetic depletion or catalytic inhibition of TTK showed significantly decreased anchorage-independent growth (Fig 3A, 3D–3F). In addition, shRNA-mediated sustained knockdown of TTK demonstrated similar trends in the inhibition of proliferation and transformed growth of PDAC cell lines. However, the magnitude of the inhibition was less pronounced than those observed following siRNA-mediated knockdown of TTK, possibly due to incomplete knockdown of TTK or isolation of stably infected cells that survived selection by compensating for the loss of TTK at the molecular level (S3 Fig).

### Override of the spindle assembly checkpoint and aberrant cell cycle progression and mitotic aberrancies and apoptosis

**Fig 4:**
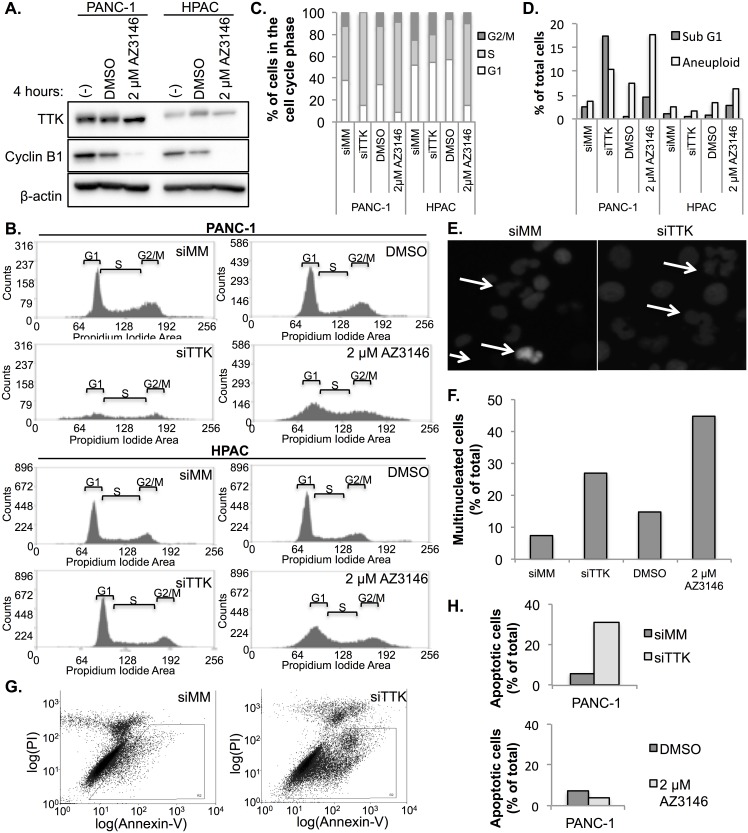
TTK inhibition overrides the SAC mediated cell cycle arrest and leads to aberrant cell cycle progression, multi-nucleation and apoptosis. (A) Immunoblot of HPAC and PANC-1 PDAC cell lines arrested in mitosis by treatment with nocodazole. Cells were then treated with 2 μM AZ3146 for 4 h and probed for expression of cyclin B1. (B) Representative flow cytometry plots of the cell cycle of HPAC and PANC-1 cell lines of 2 experiments. Cells were transfected with control or TTK targeted siRNA. 72 h post transfection cells were fixed and stained with propidium iodide. DNA content was assessed by flow cytometry. (C) Quantitation of B showing distribution of cells in each phase of the cell cycle. (D) Confocal microscopy of the PANC-1 cell line stably expressing a GFP-Histone 2B construct to visualize DNA. Chromosomal instability is visible in cells depleted of TTK in the form of multi- and micro-nucleation. (E) Quantitation of cells with multi- or micro-nucleated phenotypes. (F) Scatterplots showing induction of apoptosis with depletion of TTK. PANC-1 cells were transfected with control or a TTK targeted siRNA pool. 72 h post transfection cells were harvested and stained with the apoptotic marker Annexin V and the counterstained with propidium iodide to visualize necrotic cells. (G) quantitation of the apoptotic induction of cells used in F.

It is well established that TTK is required for proper SAC activation and function [19]. To understand the molecular mechanism whereby TTK supports PDAC growth we investigated the effects of TTK inhibition on SAC activation. HPAC and PANC-1 cells were arrested in mitosis with 100 ng/ml nocodazole and then challenged with 2 μM AZ3146. We monitored the stability of the mitotic marker cyclin B to assess silencing of the SAC. Catalytic inhibition of TTK caused a drop in the levels of cyclin B (Fig 4A), indicating that the catalytic inhibition of TTK caused an escape from checkpoint mediated mitotic arrest and accelerated mitotic progression. We next investigated the effect of checkpoint silencing on cell cycle progression. We determined the cell cycle phase distribution of HPAC and PANC-1 cells treated with siRNA or AZ3146 after 72 h by propidium iodide staining and flow cytometry analysis. Aberrant distribution of cells in each cell cycle phase occurred, specifically an increase post G2 cells, indicative of multi-nucleation (Fig 4B and 4C).

### TTK is required to prevent aneuploidy and apoptosis

Studies in other tumors have found that TTK depletion causes premature mitotic progression and often results in aberrant chromosomal segregation and aneuploidy [30,56–58]. In PDAC, TTK inhibition induced gains in the X chromosome and chromosome 17. However, the effect of TTK inhibition on the nuclear architecture is unknown. We examined the nuclear architecture of PANC-1 cells stably transduced with a lenti-viral vector encoding H2B-GFP followed by depletion of TTK using siRNA. Fluorescent microscopy revealed gross multi- and micro-nucleation in TTK depleted cell lines compared to mismatch siRNA controls, which reflected widespread chromosomal segregation defects (Fig 4D and 4E). These results demonstrate the requirement of TTK for mitotic fidelity and that depletion of TTK results in an increase of chromosomal instability and aneuploidy.

Unstable aneuploidy is often associated with cell death from mitotic catastrophe. To assess whether TTK silencing was associated with induction of apoptosis in PDAC cell lines, we monitored apoptotic induction by measuring both positive annexin-V staining and caspase-3/7 activity by flow cytometry and the Caspase-Glo 3/7 assay. siRNA mediated depletion of TTK induced apoptosis in PANC-1, Panc 10.05 and SW-1990 cells but not AsPC-1 cells. Additionally, only SW-1990 and Panc 10.05 cell lines exhibited a strong induction of apoptosis after catalytic inhibition of TTK (Fig 4F–4H, S4 Fig). These results clearly demonstrate that apoptotic induction can occur in PDAC following TTK depletion or catalytic inhibition but may be cell line dependent.

### Usp16 is a substrate of TTK

To further delineate the molecular mechanism whereby TTK regulates proper mitotic progression necessary for growth we sought to identify phosphorylation substrates of TTK. To identify putative substrates of TTK we used the Scansite3 (http://scansite.mit.edu/) prediction software and an input phosphorylation consensus motif recently identified by Hennrich et al. [59]. This motif consists of a threonine residue with acidic amino acids in the −2, and/or −3 positions and hydrophobic branched-chain amino acids (leucine, valine and isoleucine) in the +2 and +3 position [59]. Using this approach we identified 410 putative TTK phosphorylation substrates in humans. Eight of these phosphorylation substrates have known roles in cell cycle regulation (Table 3). Addition of an aspartic acid or asparagine at the -1 position of the consensus phosphorylation motif identified a single putative TTK phosphorylation substrate: ubiquitin specific peptidase 16 (Usp16, also known as Ubp-M).

**Table 3:** Predicted TTK phosphorylation substrate with known mitotic roles.

Gene Symbol	Gene Name	Function
SKA3	Spindle and kinetochore-associated protein 3	Microtubule-binding subcomplex of the outer kinetochore that is essential for proper chromosome segregation.
PDS5B	Sister chromatid cohesion protein PDS5 homolog B	Stabilizes cohesin complex association with chromatin.
USP16	Ubiquitin carboxyl-terminal hydrolase 16	Deubiquitinates H2A, one of two major ubiquitinated proteins of chromatin.
PSRC1	Proline/serine-rich coiled-coil protein 1	Required for normal congress of chromosomes at the metaphase plate, and for normal rate of chromosomal segregation during anaphase.
APC1	Anaphase-promoting complex subunit 1	Component of the anaphase promoting complex/cyclosome (APC/C), a cell cycle-regulated E3 ubiquitin ligase that controls progression through mitosis and the G1 phase of the cell cycle.
PPP2R2D	Serine/threonine-protein phosphatase 2A 55 kDa regulatory subunit B delta isoform	Regulatory subunit of PP2A holoenzyme.
KNTC1	Kinetochore-associated protein 1	Essential component of the mitotic checkpoint that prevents cells premature mitotic exit. Required for assembly of the dynein-dynactin and MAD1-MAD2 complexes onto kinetochores.
WAPL	Wings apart-like protein homolog	Regulator of sister chromatid cohesion in mitosis, which negatively regulates cohesin association with chromatin.

**Fig 5:**
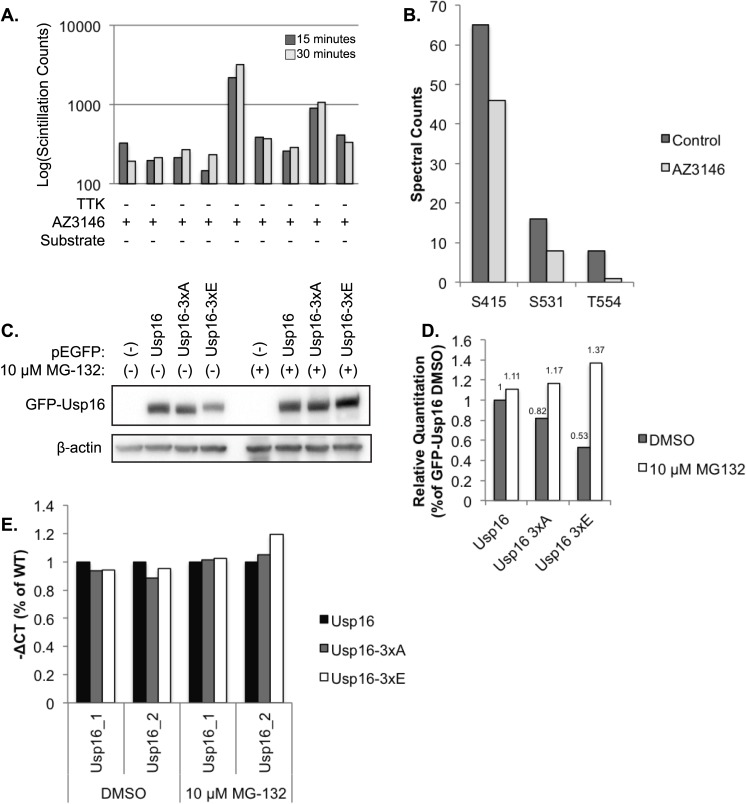
Usp16 is a TTK phosphorylation substrate. (A) *In vitro* kinase assay measuring TTK dependent phosphorylation by ^32^P incorporation measured by liquid scintillation counts. Representative of 2 independent experiments. (B) Exogenously expressed FLAG-Usp16 was immunoprecipitated from DMSO and AZ3146 treated mitotic 293FT cells, digested with trypsin and enriched for phosphopeptides. Phosphorylated residues of Usp16 were identified by mass spectrometry. Spectral counts of representative individual experiments are shown. (C) Immunoblot analysis of 293FT cells transiently transfected with control GFP, GFP-Usp16, GFP-Usp16 3xA (phosphodeficient mutant) or GFP-Usp16 3xE (phosphomimetic mutant) and treated with control DMSO or MG-132. (D) Densitometry of (C). (E) RT-PCR of Usp16 using 2 independent Taqman probes from cells used in (C), normalized to β-actin and represented as percent of WT-Usp16.

To examine if Usp16 is a direct phosphorylation substrate of TTK we performed an *in vitro* kinase assay with [^32^P]ATP and measured substrate incorporation of ^32^P. Incorporation of ^32^P on purified Usp16 was enhanced when incubated with active TTK (SignalChem) and inhibited upon addition of 2 μM AZ3146 (Fig 5A). These results indicate that Usp16 is directly phosphorylated by TTK *in vitro*. We next sought to identify the TTK dependent phosphorylation sites on Usp16 by performing mass spectrometry. TTK activity was stimulated with nocodazole in 293FT cells exogenously expressing a flag-tagged Usp16. Cells were then concurrently challenged with DMSO control or 2 μM AZ3146 for 4 h. FLAG-Usp16 from the cell lysate was purified by immunoprecipitation, digested with trypsin, and enriched for phosphopeptides. Amino acid composition of phosphopeptides was then identified by orbi-trap mass spectrometry. Analysis of the resulting spectra identified three TTK-dependent phosphorylation sites within Usp16: S415, S552, T554 (Fig 5B).

### TTK dependent phosphorylation of Usp16 causes protein degradation

To investigate the importance of phosphorylation of these three residues on Usp16 function we created a GFP tagged phospho-mimetic mutant of Usp16 where glutamic acid residues were substituted for the three identified phosphorylation sites (GFP-Usp16 3xE), and a phospho-deficient mutant of Usp16 where alanine residues were substituted for the three identified phosphorylation sites (GFP-Usp16-3xA). Immunoblot analysis of 293FT cells transfected with cDNA expression vectors encoding wild type or mutant Usp16 revealed decreased expression of the phospho-mimetic mutant of Usp16. RT-PCR with Usp16 specific primers confirmed equivalent mRNA expression, suggesting that Usp16 phosphorylation promotes protein degradation. Inhibition of the proteasome with 10 μM MG-132 restored expression of the phosphomimetic Usp16 mutant (Fig 5C–5E). Taken together, these data suggest that Usp16 is a phosphorylation substrate of TTK and that Usp16 phosphorylation on S415, S552, or T554 leads to proteasome degradation of Usp16.

## Discussion

An increased understanding of the biology of PDAC is essential to the identification of drug targets for the development of better therapies. Chromosomal instability and aneuploidy are characteristics of PDAC [8,60–64]. The SAC limits chromosomal instability by ensuring faithful segregation of sister chromatids during mitosis. Here we show RNA overexpression of the core components of the SAC in primary PDAC tumors compared to normal pancreas. We hypothesize that targeting the SAC function may alter the ability of cancer cells to adapt to aneuploidy and may be a possible therapy for PDAC.

TTK is a protein kinase required for SAC activation and was found to be overexpressed in primary PDAC [19]. *TTK* was a gene previously identified in a 25-gene signature associated with chromosomal instability and aneuploidy in cancer [56]. Thus, overexpression of TTK may represent an adaptive mechanism to sustain the growth of chromosomally unstable tumors. In support of this hypothesis, overexpression of TTK has been previously observed in multiple tumor types including PDAC [32,33], breast [24–26,65], bladder [20], esophagus [23], lung [22], anaplastic thyroid [21], and glioblastoma [30].

To investigate the role of TTK as an adaptive response to prevent excessive aneuploidy in PDAC we investigated the effect of both catalytic and genetic depletion of TTK on mitotic progression. Previous studies showed that pharmacologic inhibition of TTK catalytic activity caused an aberrant override of the SAC mediated cell cycle arrest in PDAC [32,33]. We found that this may be cell line dependent as knockdown and catalytic inhibition of TTK increased aneuploidy in the PANC-1 but not the HPAC cell line. Although this result was unexpected, previous reports have demonstrated heterogeneous response to TTK inhibition [29]. The difference in response could perhaps be explained by the hypothesis that cells with extra chromosomes may have a greater requirement for SAC function [16,32,66,67]. However, previous reported karyotypes show a modal number of 63 and 61 for the PANC-1 and HPAC cell lines respectively, suggesting that the level of aneuploidy does not account for this discrepancy [68,69]. Alternatively, the different TTK dependencies could be attributed to differences in the genetic background of the two cell lines. Previous studies have shown that aneuploidy can stimulate a p53-dependent senescence-like growth arrest [17,70,71]. PANC-1 cells have inactivating mutations in both alleles of *TP53*, whereas HPAC cells have two wild-type *TP53* alleles [72]. Mitotic errors resulting from TTK inhibition in HPAC cells may trigger p53 mediated growth arrest and prevent aneuploidy whereas PANC-1 lacking functional p53 continue to aberrantly divide. Further investigation in more PDAC cell lines with a wild-type *TP53* background will be required to determine whether TTK inhibition induces senescence-like phenotypes.

Importantly, we found that knockdown of TTK significantly decreased PDAC cell line proliferation and transformed growth in all four PDAC cell lines evaluated and that pharmacologic inhibition decreased growth in three of four cell lines. Our results are in agreement with prior reports in other tumor types where either pharmacologic inhibition or genetic knockdown of TTK resulted in a similar decrease in the growth of multiple cancer cell lines [24,26–30,32,73–75]. In addition, we observed that both transient and stable genetic depletion of TTK decreased proliferation and transformed growth, demonstrating the immediate and prolonged requirement of TTK in PDAC. One key observation from our study is the discordant response seen in the AsPC-1 cell line. Whereas genetic depletion of TTK significantly decreased growth on plastic, catalytic inhibition of TTK had no effect, suggesting that non-kinase domains of TTK may be important to support continued proliferation of these aneuploid cells. Although poorly characterized, the large non-catalytic N-terminus of TTK contains tandem tetratricopeptide repeats known to be important for protein binding [76–78]. Future studies will be required to identify binding partners and characterize how they may contribute to adapting to aneuploidy.

Apoptotic induction was observed in two of four cells lines in response to AZ3146 mediated catalytic inhibition and genetic depletion. Interestingly, we observed that 72 h catalytic inhibition of TTK with AZ3146 did not induce apoptosis in the PANC-1 cell line whereas genetic knockdown of TTK did. In contrast to our results, Slee et al. found that pharmacologic inhibition of TTK with NMS-P715 in PANC-1 cells did induce apoptosis [32]. Although PANC-1 cell lines show decrease growth in response to genetic depletion and catalytic inhibition, differences in apoptotic induction may be due to off target effects suggesting the need to further characterize these pharmacologic inhibitors of TTK. Although the AsPC-1 cell line showed decreased proliferation in response to genetic ablation, we unexpectedly observed that neither genetic depletion nor catalytic inhibition of TTK induced apoptosis in the AsPC-1 cell line. Cell death resulting from mitotic catastrophe has been previously reported without increased apoptotic induction [79]. We hypothesize that cell line specific genetic lesions may determine which cell death pathway is utilized and propose testing additional cells lines to identify biomarkers that may predict response and further characterize these pathways.

In an attempt to characterize the molecular pathways downstream of TTK, we identified Usp16 as a novel phosphorylation substrate of TTK. Usp16 dependent deubiquitination of histone H2A is a prerequisite for chromosomal condensation [17,80,81]. We identified three TTK-dependent phosphorylation sites on Usp16: S415, S552, and T554. Recently, CDK1 dependent phosphorylation of Usp16 on S552 was shown to promote an interaction between Usp16 and Plk1. This interaction results in deubiquitination of Plk1 and sustained kinetochore localization of Plk1 during metaphase that is necessary for initial kinetochore-microtubule attachment, proper chromosome alignment, and timely chromatid segregation [82]. In addition, TTK has been found to cooperate with Plk1 to regulate the SAC through phosphorylation of KNL-1 [83]. We show that point mutations in Usp16 (S415E, S552E, T554E) exhibited enhanced degradation compared to wild-type Usp16. We propose that this triple phosphorylation and subsequent degradation of Usp16 may represent another mechanism whereby TTK regulates genome stability by preventing Plk1 deubiquitination leading to its expulsion from the kinetochore, as well as preventing chromosomal condensation by inhibiting histone H2A deubiquitination, thus allowing more time to correct for errors that accumulated during DNA replication. Future studies will be required to confirm whether TTK-dependent phosphorylation of Usp16 S552 plays a similar role in regulating Plk1 to maintain proper SAC function.

The high levels of chromosomal instability that exists in PDAC present a window that may be exploited for therapy. Our results demonstrate that SAC inactivation through inhibition of the upstream activator TTK decreases the ability of PDAC to adapt and support the growth of aneuploid cells. TTK inhibition has previously been shown to enhance the radiosensitivity of human glioblastoma cells [31] as well as enhance chromosomal instability and sensitivity of cancer cell lines when combined with the microtubule targeting drugs vincristine or paclitaxel [14,29,30,84]. Consistent with this idea, the microtubule targeting nab-paclitaxel in combination with gemcitabine was approved for PDAC therapy [32,85]. Future studies will be required to determine whether TTK inhibition sensitizes PDAC cells to nab-paclitaxel. Our results support the continued need to study molecular mechanisms that allow PDAC to adapt to chromosomal instability.

## Supporting information

S1 Table Overexpressed protein kinases in primary PDAC compared to normal pancreas, with their respective fold change values and false discovery rates. (PDF)

S1 Fig Individual targeting of TTK of each siRNA used in the pool. Immunoblot analysis of HPAC and PANC-1 cell lines 48 h after transfection with 10 nM siRNA targeting TTK.
(TIF)

S2 Fig Transient TTK knockdown decreases growth in additional PDAC cell lines. (A) Immunoblot of Panc 10.05 and AsPC-1 cell lines showing protein levels of TTK in control mismatch siRNA (siMM) and a TTK siRNA (siTTK) pool 48 h after transfection. (B) Growth of Panc 10.05 and AsPC-1 PDAC cell lines transfected with control siMM and siTTK show reduced viability with TTK depletion. Cells were measured for proliferation at 48, 72, and 120 h as indicated. (C) Growth of Panc 10.05 and AsPC-1 PDAC cell lines treated with DMSO control or 2 μM AZ3146. Cells were measured for proliferation at 48, 72, and 120 h as indicated. Asterisk represent the P-value of the two-sided paired T-test (ns: P≥0.05, *:≤0.05, P **: P≤0.01, ***: P≤0.001). Results representative of at least 2 experiments.
(TIF)

S3 Fig Stable TTK knockdown decreases growth of PDAC cell lines. (A) Immunoblot of HPAC and PANC-1 cell lines showing protein levels of TTK in control mismatch shRNA (shNS) and TTK shRNA (shTTK3 and shTTK4) following infection and selection. (B) Growth of PANC-1 and HPAC cell lines infected with control shNS and two shTTK constructs show reduced viability with TTK depletion. Cells were measured for proliferation at 48, 72, and 120 h as indicated. (C) Transformed growth of PANC-1 and HPAC cell lines infected with control shNS and two shTTK constructs demonstrated reduced growth. Asterisk represent the P-value of the two-sided paired T-test (*:≤0.05, P **: P≤0.01). Results representative of at least 2 experiments.
(TIF)

S4 Fig Caspase 3/ 7activity in PDAC cell lines with TTK inhibition. (A) Immunoblot of Panc 10.05 and AsPC-1 cell lines showing protein levels of TTK in control mismatch siRNA (siMM) and a TTK siRNA (siTTK) pool 48 h after transfection. (B) Enzymatic activities of caspase 3/7 were measured in AsPC-1, SW-1990, PANC-1 and Panc 10.05 cell lines 72 hours after transfection with TTK targeting siRNA. Relative luminescence is expressed in the bar graph. (C) Enzymatic activities of caspase 3/7 were measured in AsPC-1, SW-1990, PANC-1 and Panc 10.05 cell lines 72 hours after administration of DMSO control or 2 μM AZ3146. Relative luminescence is expressed in the bar graph. Asterisk represent the P-value of the two-sided T-test paired (*:≤0.05, P **: P≤0.01, ***: P≤0.001). Results representative of at least 2 experiments.
(TIF)
