# Supplementary material for: Genetic and pharmacological inhibition of TTK impairs pancreatic cancer cell line growth by inducing lethal chromosomal instability
Source: PLoS One. 2017 Apr 5;12(4):e0174863. doi: 10.1371/journal.pone.0174863 (PMC5381904; doi:10.1371/journal.pone.0174863)
Supplement: S1 Table — (PDF) [file pone.0174863.s001.pdf]

**Supplemental Table 1. Overexpressed protein kinases in primary PDAC compared to normal pancreas, with their respective fold-change values and false discovery rates.**				
**Gene ID**	**Gene Name**	**Gene Product**	**Fold Change**	**q-value(%)**
AGI_HUM1_OLIGO_A_23_P201988	*MASTL*	microtubule associated serine/threonine kinase-like	1.75	0.53
AGI_HUM1_OLIGO_A_23_P20248	*MAP2K1*	mitogen-activated protein kinase kinase 1	1.42	2.49
AGI_HUM1_OLIGO_A_23_P207896	*CSNK1D*	casein kinase 1, delta	1.38	2.02
AGI_HUM1_OLIGO_A_23_P213114	*TEC*	tec protein tyrosine kinase	1.33	4.20
AGI_HUM1_OLIGO_A_23_P215461	*LIMK1*	LIM domain kinase 1	1.54	1.43
AGI_HUM1_OLIGO_A_23_P216920	*NEK6*	NIMA (never in mitosis gene a)-related kinase 6	1.31	5.07
AGI_HUM1_OLIGO_A_23_P24997	*CDK4*	cyclin-dependent kinase 4	1.33	2.93
AGI_HUM1_OLIGO_A_23_P251342	*GSK3B*	glycogen synthase kinase 3 beta	1.32	0.39
AGI_HUM1_OLIGO_A_23_P252106	*RIPK2*	receptor-interacting serine-threonine kinase 2	1.35	2.93
AGI_HUM1_OLIGO_A_23_P256312	*MST1R*	macrophage stimulating 1 receptor (c-met-related tyrosine kinase)	2.00	0.13
AGI_HUM1_OLIGO_A_23_P259586	*TTK*	TTK protein kinase	2.97	0.00
AGI_HUM1_OLIGO_A_23_P3204	*MAPK6*	mitogen-activated protein kinase 6	1.41	2.93
AGI_HUM1_OLIGO_A_23_P342067	*UHMK1*	U2AF homology motif (UHM) kinase 1	1.36	2.02
AGI_HUM1_OLIGO_A_23_P35219	*NEK2*	NIMA (never in mitosis gene a)-related kinase 2	3.42	0.00
AGI_HUM1_OLIGO_A_24_P166663	*CDK6*	cyclin-dependent kinase 6	1.66	1.43

**Supplemental Table 1 (continued). Overexpressed protein kinases in primary PDAC compared to normal pancreas, with their respective fold-change values and false discovery rates.**				
**Gene ID**	**Gene Name**	**Gene Product**	**Fold Change**	**q-value(%)**
AGI_HUM1_OLIGO_A_23_P359245	*MET*	met proto-oncogene (hepatocyte growth factor receptor)	2.38	0.00
AGI_HUM1_OLIGO_A_23_P39684	*TLK1*	tousled-like kinase 1	1.29	2.49
AGI_HUM1_OLIGO_A_23_P397341	*PKMYT1*	progestin and adipoQ receptor family member IV	1.79	0.00
AGI_HUM1_OLIGO_A_23_P418413	*OXSR1*	oxidative-stress responsive 1	1.33	1.12
AGI_HUM1_OLIGO_A_23_P42784	*STK31*	serine/threonine kinase 31	1.91	2.93
AGI_HUM1_OLIGO_A_23_P51646	*PLK3*	polo-like kinase 3 (Drosophila)|Tctex2 beta	1.57	5.07
AGI_HUM1_OLIGO_A_23_P55578	*RIOK3*	RIO kinase 3 (yeast)	1.48	2.49
AGI_HUM1_OLIGO_A_23_P55584	*RIOK3*	RIO kinase 3 (yeast)	1.53	1.77
AGI_HUM1_OLIGO_A_23_P56978	*PTK6*	PTK6 protein tyrosine kinase 6	3.21	0.00
AGI_HUM1_OLIGO_A_23_P57667	*PLXNA1*	plexin A1	1.71	0.00
AGI_HUM1_OLIGO_A_23_P66732	*GSG2*	germ cell associated 2 (haspin)	1.44	2.49
AGI_HUM1_OLIGO_A_23_P75989	*PAK1*	p21/Cdc42/Rac1-activated kinase 1 (STE20 homolog, yeast)	1.49	2.02
AGI_HUM1_OLIGO_A_23_P76731	*RAGE*	renal tumor antigen	1.52	2.02
AGI_HUM1_OLIGO_A_23_P94422	*MELK*	maternal embryonic leucine zipper kinase	1.80	0.53

**Supplemental Table 1 (continued). Overexpressed protein kinases in primary PDAC compared to normal pancreas, with their respective fold-change values and false discovery rates.**				
**Gene ID**	**Gene Name**	**Gene Product**	**Fold Change**	**q-value(%)**
AGI_HUM1_OLIGO_A_24_P251899	*CSNK1A1*	casein kinase 1, alpha 1	1.30	3.47
AGI_HUM1_OLIGO_A_24_P313504	*PLK1*	endoplasmic reticulum to nucleus signaling 2|polo-like kinase 1 (Drosophila)	1.52	2.02
AGI_HUM1_OLIGO_A_24_P319613	*NEK2*	NIMA (never in mitosis gene a)-related kinase 2	2.14	0.00
AGI_HUM1_OLIGO_A_24_P333663	*MAPK6*	mitogen-activated protein kinase 6	1.45	3.47
AGI_HUM1_OLIGO_A_24_P37441	*PDK1*	pyruvate dehydrogenase kinase, isozyme 1	1.76	0.53
AGI_HUM1_OLIGO_A_24_P42603	*TRIO*	triple functional domain (PTPRF interacting)	1.43	5.07
AGI_HUM1_OLIGO_A_24_P830690	*PDPK1*	3-phosphoinositide dependent protein kinase-1	1.36	4.20
AGI_HUM1_OLIGO_A_24_P94054	*STK4*	serine/threonine kinase 4	1.51	4.20
AGI_HUM1_OLIGO_A_32_P140501	*AXL*	AXL receptor tyrosine kinase	1.49	5.07
AGI_HUM1_OLIGO_A_32_P25204	*PRKDC*	similar to protein kinase, DNA-activated, catalytic polypeptide	1.35	3.47
AGI_HUM1_OLIGO_A_32_P62997	*PBK*	PDZ binding kinase	2.76	0.00
AGI_HUM1_OLIGO_A_32_P119174	*IPPK*	inositol 1,3,4,5,6-pentakisphosphate 2-kinase|centromere protein P	1.37	5.07
AGI_HUM1_OLIGO_A_24_P245646	*TP53RK*	TP53 regulating kinase	1.30	3.47

**Supplemental Table 1 (continued). Overexpressed protein kinases in primary PDAC compared to normal pancreas, with their respective fold-change values and false discovery rates.**				
**Gene ID**	**Gene Name**	**Gene Product**	**Fold Change**	**q-value(%)**
AGI_HUM1_OLIGO_A_24_P76319	*LOC642609*	similar to tau tubulin kinase 2	1.44	5.07
AGI_HUM1_OLIGO_A_23_P10559	*AATK*	apoptosis-associated tyrosine kinase	-1.98	1.77
AGI_HUM1_OLIGO_A_23_P110791	*CSF1R*	colony stimulating factor 1 receptor, formerly McDonough feline sarcoma viral (v-fms) oncogene homolog	-1.89	4.20
AGI_HUM1_OLIGO_A_23_P125596	*RPS6KA6*	ribosomal protein S6 kinase, 90kDa, polypeptide 6	-1.28	4.20
AGI_HUM1_OLIGO_A_23_P126416	*TIE1*	tyrosine kinase with immunoglobulin-like and EGF-like domains 1	-1.73	1.43
AGI_HUM1_OLIGO_A_23_P128447	*LRRK2*	leucine-rich repeat kinase 2	-1.64	0.22
AGI_HUM1_OLIGO_A_23_P134125	*MAP3K5*	mitogen-activated protein kinase kinase kinase 5	-1.41	3.47
AGI_HUM1_OLIGO_A_23_P142304	*MKNK2*	MAP kinase interacting serine/threonine kinase 2	-1.74	2.93
AGI_HUM1_OLIGO_A_23_P142310	*MKNK2*	MAP kinase interacting serine/threonine kinase 2	-1.61	2.02
AGI_HUM1_OLIGO_A_23_P147711	*NPR1*	natriuretic peptide receptor A/guanylate cyclase A (atrionatriuretic peptide receptor A)	-1.75	0.14
AGI_HUM1_OLIGO_A_23_P159169	*AATK*	apoptosis-associated tyrosine kinase	-1.29	3.47
AGI_HUM1_OLIGO_A_23_P164057	*MAPK7*	microfibrillar-associated protein 4|mitogen-activated protein kinase 7	-2.40	0.53
AGI_HUM1_OLIGO_A_23_P16817	CLK1	CDC-like kinase 1	-1.67	1.12

**Supplemental Table 1 (continued). Overexpressed protein kinases in primary PDAC compared to normal pancreas, with their respective fold-change values and false discovery rates.**				
**Gene ID**	**Gene Name**	**Gene Product**	**Fold Change**	**q-value(%)**
AGI_HUM1_OLIGO_A_23_P202245	*RET*	ret proto-oncogene	-1.46	5.07
AGI_HUM1_OLIGO_A_23_P169819	*EPHA3*	EPH receptor A3	-1.87	2.02
AGI_HUM1_OLIGO_A_23_P205900	*NTRK3*	neurotrophic tyrosine kinase, receptor, type 3	-1.91	0.27
AGI_HUM1_OLIGO_A_23_P207517	*PDK2*	pyruvate dehydrogenase kinase, isozyme 2	-1.36	1.77
AGI_HUM1_OLIGO_A_23_P211985	*SNRK*	SNF related kinase	-1.60	1.77
AGI_HUM1_OLIGO_A_23_P219105	*FGFR1*	fibroblast growth factor receptor 1 (fms-related tyrosine kinase 2, Pfeiffer syndrome)	-1.46	3.47
AGI_HUM1_OLIGO_A_23_P253602	*BMX*	BMX non-receptor tyrosine kinase	-1.81	1.12
AGI_HUM1_OLIGO_A_23_P300033	*PDGFRA*	platelet-derived growth factor receptor, alpha polypeptide	-2.04	2.02
AGI_HUM1_OLIGO_A_23_P301304	*FGFR1*	fibroblast growth factor receptor 1 (fms-related tyrosine kinase 2, Pfeiffer syndrome)	-1.75	1.43
AGI_HUM1_OLIGO_A_23_P34804	*NTRK1*	neurotrophic tyrosine kinase, receptor, type 1	-1.55	4.20
AGI_HUM1_OLIGO_A_23_P372923	*FGFR1*	fibroblast growth factor receptor 1 (fms-related tyrosine kinase 2, Pfeiffer syndrome)	-1.47	5.07
AGI_HUM1_OLIGO_A_23_P374695	*TEK*	TEK tyrosine kinase, endothelial (venous malformations, multiple cutaneous and mucosal)	-2.89	0.00
AGI_HUM1_OLIGO_A_23_P397455	*ACVR1C*	activin A receptor, type IC	-1.67	5.07

**Supplemental Table 1 (continued). Overexpressed protein kinases in primary PDAC compared to normal pancreas, with their respective fold-change values and false discovery rates.**				
**Gene ID**	**Gene Name**	**Gene Product**	**Fold Change**	**q-value(%)**
AGI_HUM1_OLIGO_A_23_P46618	*PLXNA2*	plexin A2	-1.94	3.47
AGI_HUM1_OLIGO_A_23_P55107	*ULK2*	unc-51-like kinase 2 (C. elegans)	-1.82	0.27
AGI_HUM1_OLIGO_A_23_P424	*MARK1*	MAP/microtubule affinity-regulating kinase 1	-1.64	2.02
AGI_HUM1_OLIGO_A_23_P61674	*CLK4*	CDC-like kinase 4	-1.56	2.02
AGI_HUM1_OLIGO_A_23_P84974	*NRK*	Nik related kinase	-2.27	3.47
AGI_HUM1_OLIGO_A_24_P106112	*PKD2*	polycystic kidney disease 2 (autosomal dominant)	-1.41	4.20
AGI_HUM1_OLIGO_A_24_P179585	*MARK1*	MAP/microtubule affinity-regulating kinase 1	-1.47	4.20
AGI_HUM1_OLIGO_A_24_P243749	*PDK4*	pyruvate dehydrogenase kinase, isozyme 4	-3.69	0.00
AGI_HUM1_OLIGO_A_24_P263144	*BMX*	BMX non-receptor tyrosine kinase	-1.98	0.22
AGI_HUM1_OLIGO_A_24_P319923	*MYLK*	myosin, light chain kinase	-2.25	4.20
AGI_HUM1_OLIGO_A_24_P4171	*FGFR1*	fibroblast growth factor receptor 1 (fms-related tyrosine kinase 2, Pfeiffer syndrome)	-1.39	4.20
AGI_HUM1_OLIGO_A_24_P71973	*KDR*	kinase insert domain receptor (a type III receptor tyrosine kinase)	-1.68	1.77
AGI_HUM1_OLIGO_A_32_P100379	*PDGFRA*	platelet-derived growth factor receptor, alpha polypeptide	-1.79	0.39

**Supplemental Table 1 (continued). Overexpressed protein kinases in primary PDAC compared to normal pancreas, with their respective fold-change values and false discovery rates.**				
**Gene ID**	**Gene Name**	**Gene Product**	**Fold Change**	**q-value(%)**
AGI_HUM1_OLIGO_A_32_P105865	*ROCK1*	Rho-associated, coiled-coil containing protein kinase 1	-1.60	5.07
AGI_HUM1_OLIGO_A_32_P183765	*ERBB4*	v-erb-a erythroblastic leukemia viral oncogene homolog 4 (avian)	-2.13	3.47
AGI_HUM1_OLIGO_A_32_P200586	*CLK1*	CDC-like kinase 1	-1.37	3.47
AGI_HUM1_OLIGO_A_32_P94160	*PRKAA2*	protein kinase, AMP-activated, alpha 2 catalytic subunit	-1.60	2.49
