# Supplementary figures and images for: Genetic and pharmacological inhibition of TTK impairs pancreatic cancer cell line growth by inducing lethal chromosomal instability
Source: PLoS One. 2017 Apr 5;12(4):e0174863. doi: 10.1371/journal.pone.0174863 (PMC5381904; doi:10.1371/journal.pone.0174863)

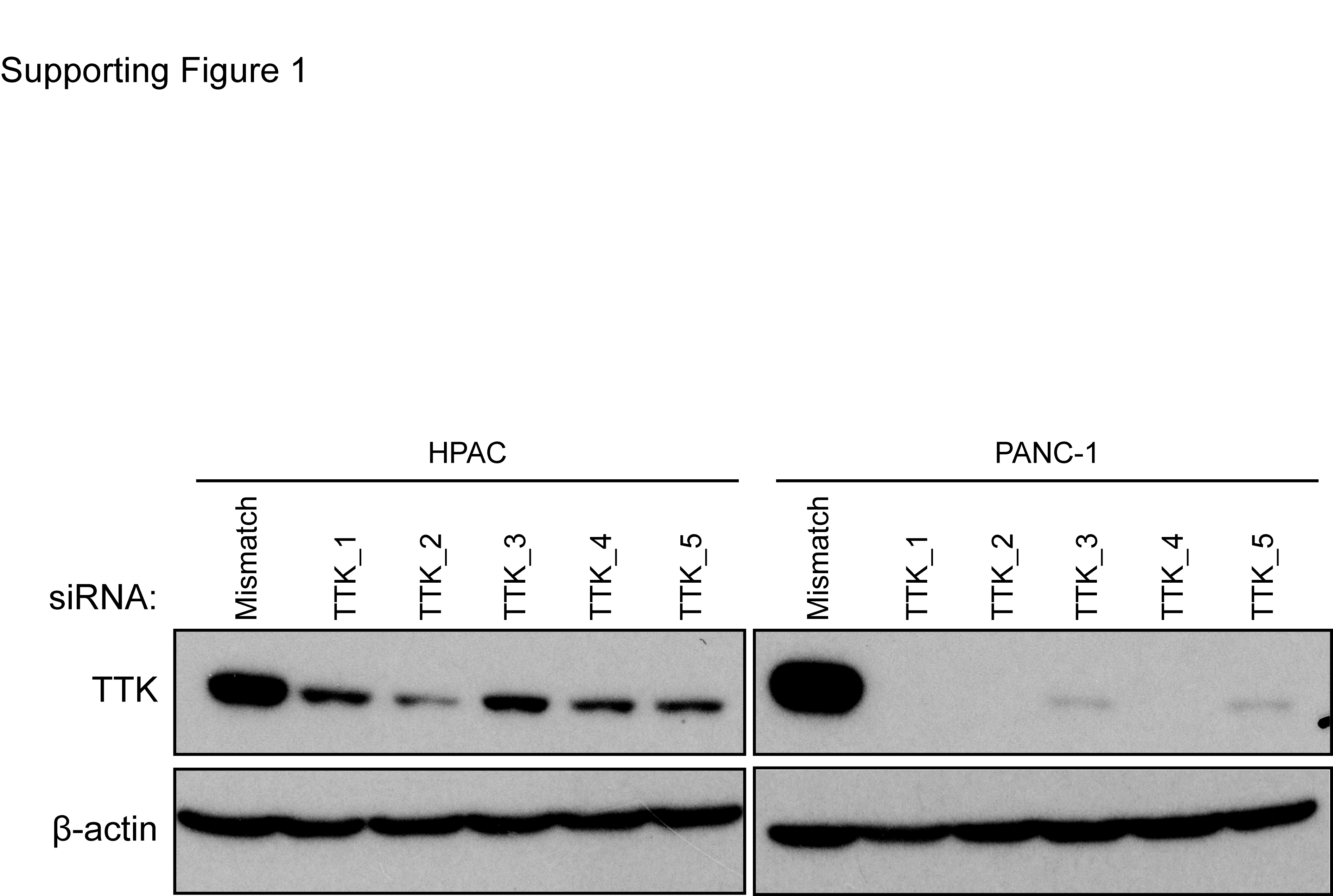

Supplement: S1 Fig — Immunoblot analysis of HPAC and PANC-1 cell lines 48 h after transfection with 10 nM siRNA targeting TTK. (TIF) [file pone.0174863.s002.tif]

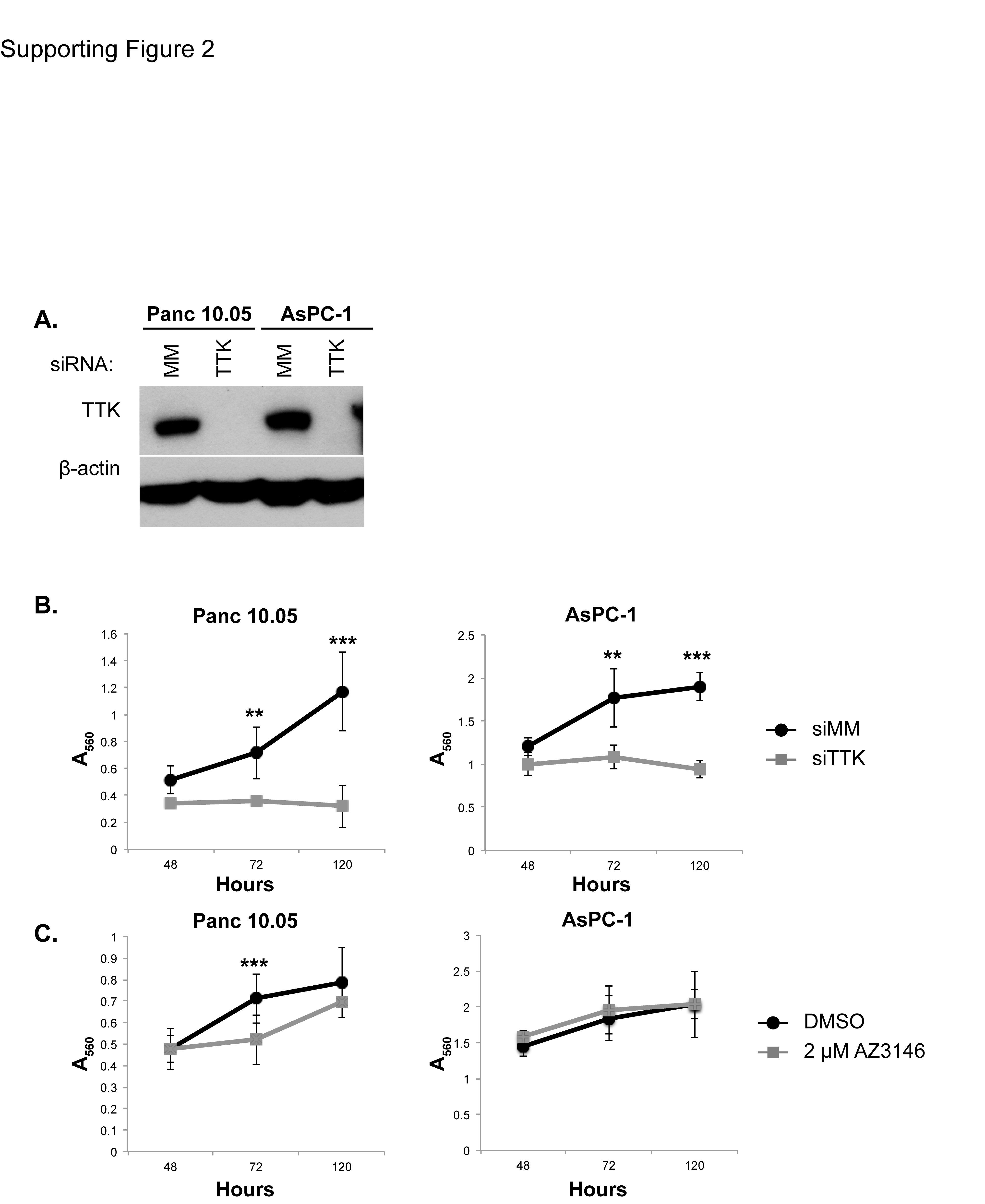

Supplement: S2 Fig — (A) Immunoblot of Panc 10.05 and AsPC-1 cell lines showing protein levels of TTK in control mismatch siRNA (siMM) and a TTK siRNA (siTTK) pool 48 h after transfection. (B) Growth of Panc 10.05 and AsPC-1 PDAC cell lines transfected with control siMM and siTTK show reduced viability with TTK depletion. Cells were measured for proliferation at 48, 72, and 120 h as indicated. (C) Growth of Panc 10.05 and AsPC-1 PDAC cell lines treated with DMSO control or 2 μM AZ3146. Cells were measured for proliferation at 48, 72, and 120 h as indicated. Asterisk represent the P-value of the two-sided paired T-test (ns: P≥0.05, *:≤0.05, P **: P≤0.01, ***: P≤0.001). Results representative of at least 2 experiments. (TIF) [file pone.0174863.s003.tif]

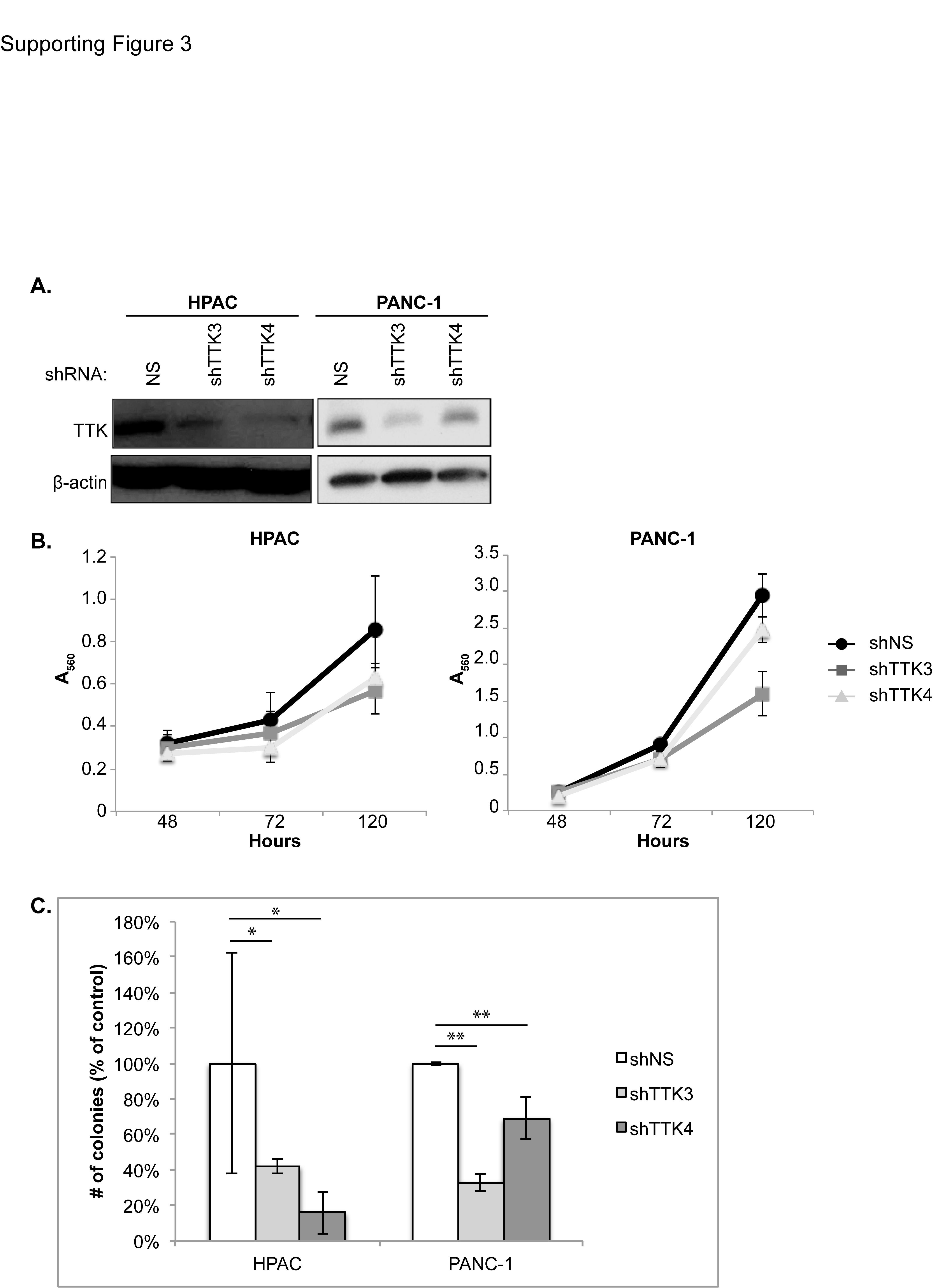

Supplement: S3 Fig — (A) Immunoblot of HPAC and PANC-1 cell lines showing protein levels of TTK in control mismatch shRNA (shNS) and TTK shRNA (shTTK3 and shTTK4) following infection and selection. (B) Growth of PANC-1 and HPAC cell lines infected with control shNS and two shTTK constructs show reduced viability with TTK depletion. Cells were measured for proliferation at 48, 72, and 120 h as indicated. (C) Transformed growth of PANC-1 and HPAC cell lines infected with control shNS and two shTTK constructs demonstrated reduced growth. Asterisk represent the P-value of the two-sided paired T-test (*:≤0.05, P **: P≤0.01). Results representative of at least 2 experiments. (TIF) [file pone.0174863.s004.tif]

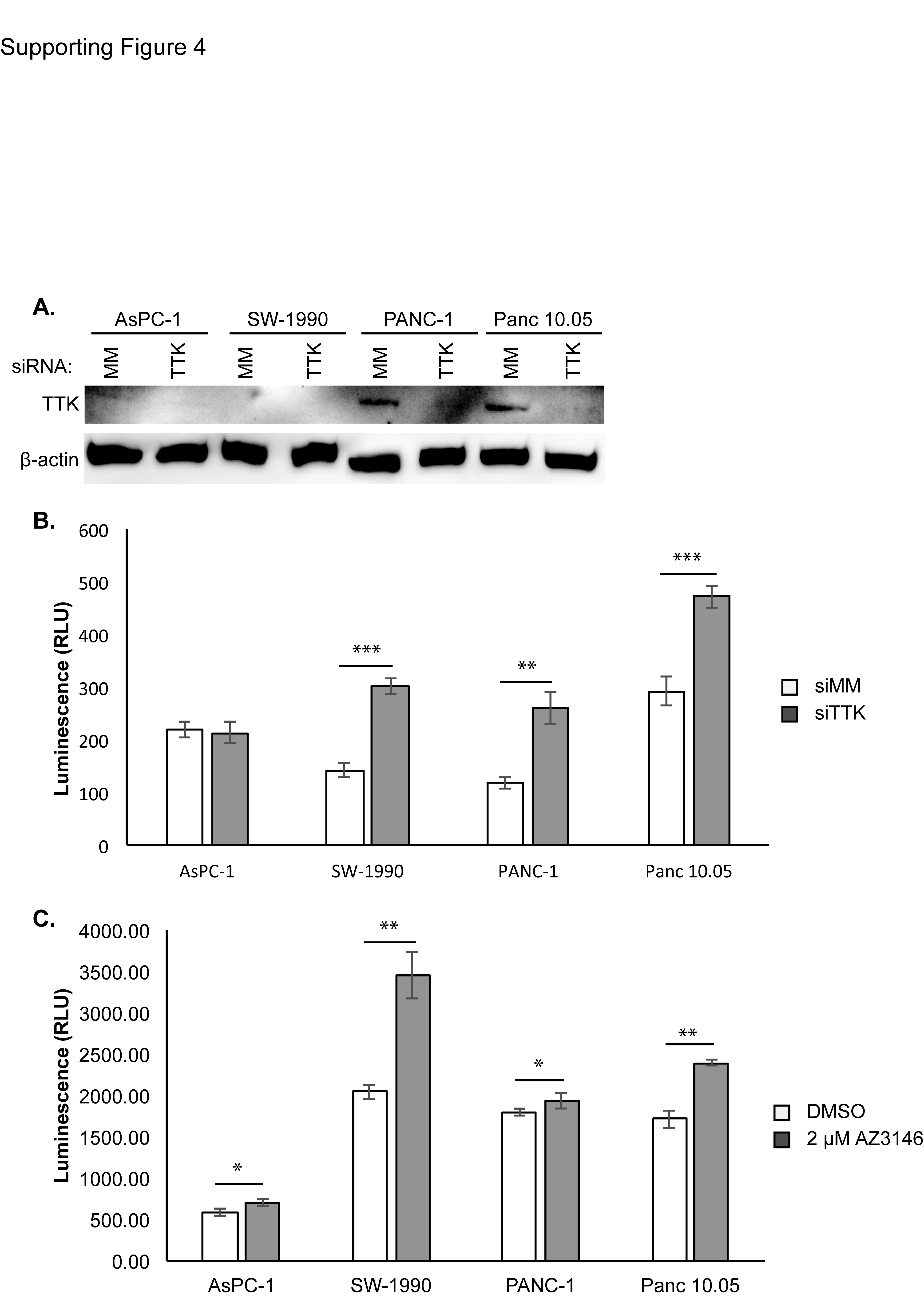

Supplement: S4 Fig — (A) Immunoblot of Panc 10.05 and AsPC-1 cell lines showing protein levels of TTK in control mismatch siRNA (siMM) and a TTK siRNA (siTTK) pool 48 h after transfection. (B) Enzymatic activities of caspase 3/7 were measured in AsPC-1, SW-1990, PANC-1 and Panc 10.05 cell lines 72 hours after transfection with TTK targeting siRNA. Relative luminescence is expressed in the bar graph. (C) Enzymatic activities of caspase 3/7 were measured in AsPC-1, SW-1990, PANC-1 and Panc 10.05 cell lines 72 hours after administration of DMSO control or 2 μM AZ3146. Relative luminescence is expressed in the bar graph. Asterisk represent the P-value of the two-sided T-test paired (*:≤0.05, P **: P≤0.01, ***: P≤0.001). Results representative of at least 2 experiments. (TIF) [file pone.0174863.s005.tif]
